# GABriella, a Phase 2a study investigating the gamma‐secretase modulator RG6289 in individuals at risk for and at the prodromal stage of Alzheimer’s disease: baseline characteristics of the recruited participants

**DOI:** 10.1002/alz70861_108331

**Published:** 2025-12-23

**Authors:** Rosanna Tortelli, Enrique Gaspar, Irma T Kurniawan, Petr Novak, Christoph Wandel, Tianxu Yang, Lisa Squassante, Archana Nair, Tayo Bodede, Julie Cook, Natasha O'Sullivan, Ana Maria Buehlmann, Stefan De Buck, Thomas Mueggler, Luka Kulic, Geoffrey A. Kerchner, Irene Gerlach

**Affiliations:** ^1^ Hoffmann‐La Roche Ltd, Basel, Basel Switzerland; ^2^ Hoffmann‐La Roche Ltd, Beijing, Beijing China; ^3^ Hoffmann‐La Roche Ltd, Welwyn, Welwyn UK; ^4^ F. Hoffmann‐La Roche Ltd, Basel, Basel Switzerland

## Abstract

**Background:**

RG6289 is a novel, potent and selective oral γ‐secretase modulator (GSM) in development for Alzheimer’s disease (AD). In the Entry‐into‐Human study in healthy volunteers, RG6289 demonstrated a favorable safety and tolerability profile, along with the anticipated pharmacodynamic effect of reducing amyloidogenic Aβ_42_ and increasing the shorter, non‐amyloidogenic isoforms Aβ_38_ and Aβ_37_ in both cerebrospinal fluid and plasma. This abstract presents the baseline characteristics of the population recruited into the ongoing GABriella Ph2a study.

**Method:**

GABriella is a randomized, double blind, placebo‐controlled, multi‐center, parallel group, dose‐range finding study investigating the safety, tolerability and the effect of RG6289 on key AD‐related biomarkers. The study includes individuals aged 60 to 85 years who are amyloid positive (≥24 centiloids on amyloid PET) and either cognitively unimpaired (CU) or with mild cognitive impairment (MCI) due to AD, diagnosed according to the NIA‐AA research framework criteria 2018. Clinical Dementia Rating Scale ‐ global score (CDR‐GS) at screening can be either 0 or 0.5. The trial includes three dose levels and one placebo arm and aims to recruit approximately 245 individuals who will be treated for 72 weeks.

**Result:**

As of April 11th 2025, 96% of the targeted participant number has been randomized, with a mean age of 72 years (SD: 5) and a mean BMI of 25,1 (SD: 3,5). 55% are females. 15% are cognitively unimpaired, and 85% have a diagnosis of MCI due to AD. 9% have a CDR‐GS of 0, and 91% have a CDR‐GS of 0.5. 36% are on anticholinesterase standard‐of‐care therapy. The mean amyloid load at baseline is 76.8 centiloids (SD: 25.5). Recruitment is expected to be completed shortly.

**Conclusion:**

GABriella is the first clinical trial assessing the effects of a brain‐penetrant, potent, and selective GSM in amyloid‐positive individuals, who are either CU or have MCI due to AD. This study will provide critical insights into the safety, tolerability, and pharmacodynamic effects of RG6289 on key AD‐related biomarkers. The results will contribute to understanding the potential of GSM therapy in the early stages of AD.

